# Probabilistic landscape of seizure semiology localizing values

**DOI:** 10.1093/braincomms/fcac130

**Published:** 2022-05-19

**Authors:** Ali Alim-Marvasti, Gloria Romagnoli, Karan Dahele, Hadi Modarres, Fernando Pérez-García, Rachel Sparks, Sébastien Ourselin, Matthew J. Clarkson, Fahmida Chowdhury, Beate Diehl, John S. Duncan

**Affiliations:** 1 Department of Clinical and Experimental Epilepsy, Queen Square Institute of Neurology, UCL, London, UK; 2 Department of Medical Physics and Biomedical Engineering, UCL, London, UK; 3 Wellcome/EPSRC Centre for Interventional and Surgical Sciences (WEISS), London, UK; 4 National Hospital for Neurology and Neurosurgery, London, UK; 5 Nuffield Department of Clinical Neurosciences, University of Oxford, Oxford, UK; 6 University College London Medical School, London, UK; 7 Faculty of Engineering, University of Cambridge, Cambridge, UK; 8 School of Biomedical Engineering & Imaging Sciences, King’s College London, London, UK

**Keywords:** phenotype, data-driven, cortical localization, epilepsy surgery, presurgical

## Abstract

Semiology describes the evolution of symptoms and signs during epileptic seizures and contributes to the evaluation of individuals with focal drug-resistant epilepsy for curative resection. Semiology varies in complexity from elementary sensorimotor seizures arising from primary cortex to complex behaviours and automatisms emerging from distributed cerebral networks. Detailed semiology interpreted by expert epileptologists may point towards the likely site of seizure onset, but this process is subjective. No study has captured the variances in semiological localizing values in a data-driven manner to allow objective and probabilistic determinations of implicated networks and nodes. We curated an open data set from the epilepsy literature, in accordance with Preferred Reporting Items for Systematic Reviews and Meta-Analyses guidelines, linking semiology to hierarchical brain localizations. A total of 11 230 data points were collected from 4643 patients across 309 articles, labelled using ground truths (postoperative seizure-freedom, concordance of imaging and neurophysiology, and/or invasive EEG) and a designation method that distinguished between semiologies arising from a predefined cortical region and descriptions of neuroanatomical localizations responsible for generating a particular semiology. This allowed us to mitigate temporal lobe publication bias by filtering studies that preselected patients based on prior knowledge of their seizure foci. Using this data set, we describe the probabilistic landscape of semiological localizing values as forest plots at the resolution of seven major brain regions: temporal, frontal, cingulate, parietal, occipital, insula, and hypothalamus, and five temporal subregions. We evaluated the intrinsic value of any one semiology over all other ictal manifestations. For example, epigastric auras implicated the temporal lobe with 83% probability when not accounting for the publication bias that favoured temporal lobe epilepsies. Unbiased results for a prior distribution of cortical localizations revised the prevalence of temporal lobe epilepsies from 66% to 44%. Therefore, knowledge about the presence of epigastric auras updates localization to the temporal lobe with an odds ratio (OR) of 2.4 [CI_95%_ (1.9, 2.9); and specifically, mesial temporal structures OR: 2.8 (2.3, 2.9)], attesting the value of epigastric auras. As a further example, although head version is thought to implicate the frontal lobes, it did not add localizing value compared with the prior distribution of cortical localizations [OR: 0.9 (0.7, 1.2)]. Objectification of the localizing values of the 12 most common semiologies provides a complementary view of brain dysfunction to that of lesion-deficit mappings, as instead of linking brain regions to phenotypic-deficits, semiological phenotypes are linked back to brain sources. This work enables coupling of seizure propagation with ictal manifestations, and clinical support algorithms for localizing seizure phenotypes.

## Teaser

Alim-Marvasti et al. curated Semio2Brain, the largest data-driven and open data set from a systematic review of the epilepsy literature, linking semiology to brain regions. About 11 230 data points were collected from 4643 patients across 309 articles. The absolute and relative probabilistic cerebral localizing values of seizure semiology are presented.

## Graphical Abstract

**Graphical abstract:**
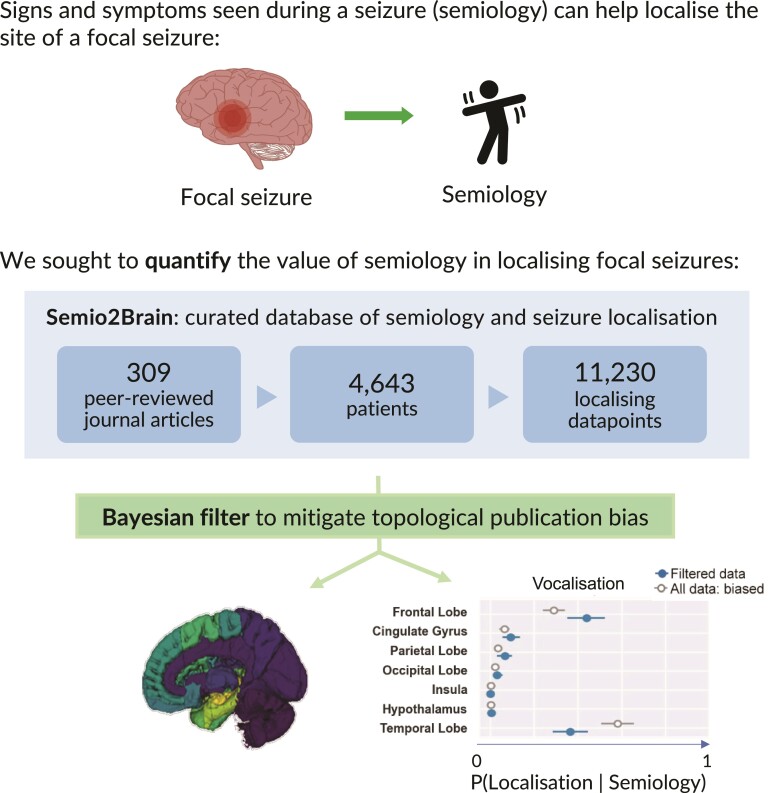

## Introduction

Seizure semiology is the chronological evolution of the symptoms and signs manifested during an epileptic seizure. It is integral to a wide variety of clinical assessments, including the evaluation of the degree of seizure focality,^1,2^ the multi-dimensional and multi-axial diagnoses of epilepsy,^3,4^ and the International League Against Epilepsy (ILAE) classification system.^5^ Semiological analysis is a vital but time-consuming element in the presurgical assessment of patients with focal drug-resistant epilepsy (fDRE) to localize seizure foci.^6^

Semiology varies from elementary sensorimotor seizures that follow a neuroanatomical homunculus, to complex behaviours, and automatisms emerging from distributed network activity in the brain. Complex semiology is thought to arise from combinations of activations and inhibitions in disparate networks involving associative cortex.^7,8^ Chronological evolution depends on network connectivity, and brain regions physically distal to the seizure-onset zone can be involved earlier in the sequence than adjacent brain regions.^9^

The role of semiology in the presurgical assessment of individuals with fDRE is often limited to the localization of the symptomatogenic zone which for simple semiology is the brain region directly responsible, but the seizure-onset zone may be distant and symptomatically silent, and so concordance is sought with neuroimaging and neurophysiology for the estimation of the seizure-onset zone. Nearly 15 million patients worldwide have fDRE, and surgery can be curative by excising the epileptogenic zone, which by definition is the smallest region of brain (assumed to contain the seizure-onset zone) that when resected renders the patient seizure-free.^10–12^ The site of seizure onset may be silent and located at a distance to the symptomatogenic zone. The role of semiology has therefore been limited to indirectly determining the epileptogenic zone via the symptomatogenic zone.^13^

There is a vast literature on seizure semiology, starting in the modern era with Hughlings Jackson.^14^ There have been numerous reviews on the localizing values of single semiologies^6,15^ and some have also investigated sequences of semiologies.^16^ Individual studies have however been restricted to small samples of patients with inadequate ground truths, sometimes with contradictory findings such as unilateral upper limb automatisms having ipsilateral seizure onsets or no lateralizing value.^14,15,17,18^ Although some studies suggest that good detailed semiology is probably as good as scalp-EEG and MRI for localization,^19^ no definitive attempt has been made to summarize the literature in a data-driven way to enable objective determination of localizing values. There are several reasons for this. First, although the literature is vast, adequately large single-centre data are scarce. Second, inadequate ground truths have led to the localizing value of a semiology being based on expert opinion about its perceived symptomatogenic zone, and this circular logic has been promulgated by machine learning models that use semiology to predict the epileptogenic zone.^20^ Third, there have been changes in semiological terminology and classifications over time, and different centres have used divergent or inconsistent terms. For example, whereas head turn and head version have previously been used interchangeably, the former is currently used to indicate unforced head turns while the latter describes forced deviation of the head as if to look over the shoulder, typically with the chin turned upward. Fourth, there is a known but hitherto unmeasured publication bias in favour of temporal lobe epilepsy (TLE) surgeries, which carry the best outcomes and are performed most often, potentially biasing localizing values, as semiologies that are relatively rare for TLE in generative models, may nevertheless be reported more frequently in TLE.

Lesion-deficit mappings have informed neuroscience about the hierarchical structure and function of the brain. A destructive lesion, such as a stroke, can result in permanent deficits in function. Tools such as voxel-based lesion-symptom mappings exist for evaluating statistical relationships between damage to specific brain regions and resulting deficits.^21^ Seizure semiology localizing values are the double-inverse: (i) instead of loss of function from a lesion, the seizure-onset zone generates epileptogenic high-frequency oscillations^22^ that manifest as seizure semiology; and (ii) instead of linking brain regions to symptom deficits, semiology is linked to brain regions. Our understanding of the hierarchical function of the brain could therefore be complemented by quantifying semiological localizing values.

Although the clinical value of any particular semiology can in theory be evaluated by Bayesian-belief elicitation of expert epileptologists, in the absence of grounded objectives, responses would capture subjective values.^23^ Here we introduce the largest ever database to evaluate semiological localizing values objectively, using ground truths that do not rely on semiology or the symptomatogenic zone itself, with data-driven and Bayesian methods to evaluate and mitigate publication bias. We use a semiological taxonomy replacement that can adapt to future changes in terminology to query the database. We use the earliest reported semiology, where available, rather than the chronological sequence of semiologies, as chronological sequence data are not readily available and the subset of brain regions involved in the early production and propagation of semiology, the ‘early spread network’, are more tightly linked to networks constituting the epileptogenic zone than semiology occurring as a result of seizure propagation.^7^

We hypothesized that a systematic, data-driven review of the literature could describe the probabilistic landscape of semiological localizing values at the resolution of seven major cortical regions and five temporal subregions and be used to evaluate the relative value of any one semiology over all other ictal manifestations.

## Methods

### Methods overview

We curated a large database from a systematic review of the epilepsy literature on seizure semiology localizations based on three ground truths. We used a taxonomy of equivalent terms to categorize the collected semiologies and brain localizations then queried the database to ascertain the probabilistic value that a semiology localized to each brain region.

To mitigate the publication bias from the systematic review that favoured temporal lobe epilepsies, during data collection we labelled semiology localization data as arising from either topological or non-topological studies. Topological studies (TS) were those that focused on a specific localization e.g. temporal lobe, whereas non-TS focused on the semiology.

Separately, we determined the overall distribution of all brain localizations in the database and mitigated for publication bias using non-TS to arrive at our best estimate for an unbiased distribution of localizations (EUD-Locs). Using this, we calculated the relative odds ratio (OR) of a semiology localizing to a specific brain region compared with all other semiologies.

### Semio2Brain database

**Figure 1:**
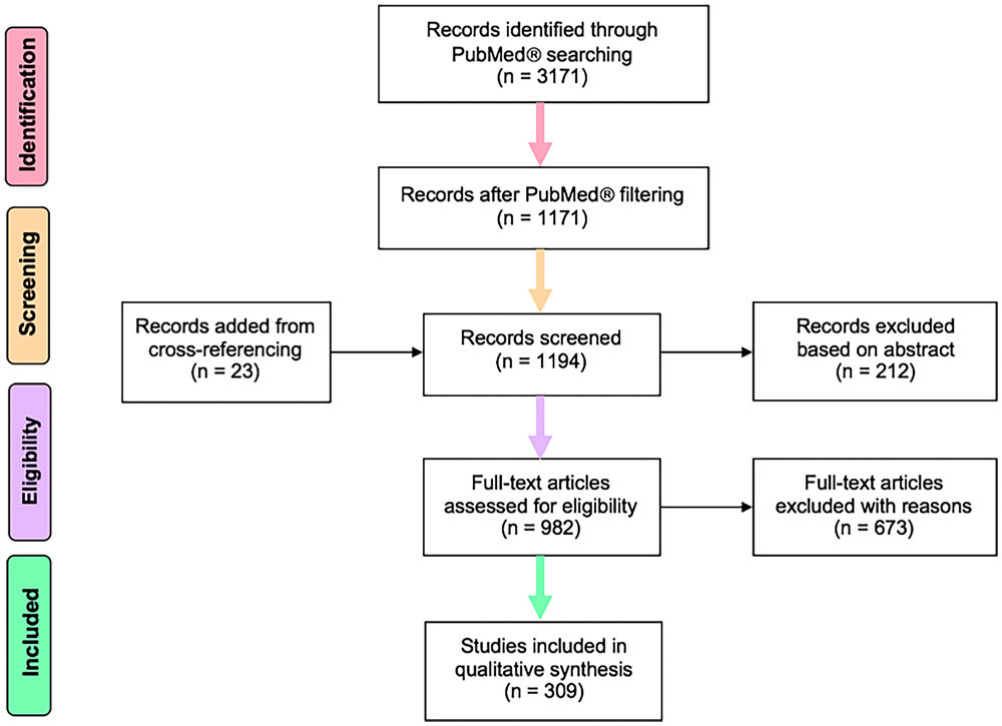
**PRISMA flow diagram.** Of the included studies, 23 were in Spanish, 11 in French, 8 in German, and the rest in English. Two hundred and twenty of 1171 were review articles. (Adapted from Moher et al. The PRISMA Statement 2009.)

We curated a unique open-access database that links semiology to brain localizations (Semio2Brain v.1.2.2, 2021, doi:10.5281/zenodo.4473240) based on a systematic review of the research literature in accordance with Preferred Reporting Items for Systematic Reviews and Meta-Analyses (PRISMA) guidelines.^24^ Data were extracted from 309 articles that met inclusion and exclusion criteria by two independent researchers (neurologist and post-doctoral researcher) (Fig. 1). Search terms, inclusion, and exclusion criteria are in Supplementary Methods.

*Semio2Brain* database has the following structure:

#### Seizure semiology

When described, the earliest reported semiology of patients with epilepsy were collected, otherwise the list of semiologies the patient had, using the exact wording of the descriptions as reported in the literature (e.g. ‘right arm flexed and left arm extended’) and summarized using a glossary of descriptive semiological categories at the point of data collection, where possible according to the ILAE Task Force on Classification and Terminology (e.g. ‘left asymmetric tonic’).^25^

During data collection, some studies described detailed semiological evolution, making it clear which epileptic symptom or sign occurred first. In these circumstances the initial semiology was collected along with its ground truth localization. Most studies, however, reported the list of semiologies or focused on a single one without clarifying where it occurred in the sequence of semiology—especially as many individuals reported in the literature had more than one seizure type with variable evolutions—in these cases the list of all semiologies were collected along with the final ground truth localization.

#### Lateralizing data points

The laterality of the semiology and/or the patients’ dominant hemisphere were determined. Laterality data points were collected relative to the semiology as ipsilateral or contralateral; and relative to hemispheric language dominance as dominant or non-dominant. *Semio2Brain* datapoint entries were at the level of individual patient semiologies.

#### Hierarchical brain regions

Hierarchical brain categories were devised, the top-level being temporal, frontal, parietal, occipital, cingulate, insula, hypothalamus, and cerebellum. The anatomical hierarchy was iteratively developed based on clinical descriptions of cortical localization during data collection, resulting in a total of 103 descriptive regions-of-interest. Each localizing semiology from a patient was multi-one-hot encoded, such that the number of localizing data points for a semiology was greater than or equal to the number of patients with that semiology.

There were separate standalone (non-hierarchical) categories for the subcallosal cortex, sulci and interlobar junctions: frontotemporal, temporo-occipital, temporo-parietal, fronto-temporo-parietal, temporo-parieto-occipital, parieto-occipital, fronto-parietal, and perisylvian. The interlobar junction categories were devised only to make the data entry process more efficient, instead of individually entering data in a hierarchical manner across several lobes and their subregions. Prior to data analysis, we redistributed these to their appropriate top-level localizations and subregions programmatically (Supplementary Methods).

#### Ground truths

Only semiology from patients with the following ground truths were collected:

- ‘seizure-freedom’: had epilepsy surgery and remained seizure-free for at least 12 months (Engel Ia or Ib, or ILAE 1 or 2, or Engel I if not otherwise specified but not worse than Engel Ib or ILAE 2)
- ‘concordance’: concordant imaging and electrophysiology, which included mostly MRI and (ictal or interictal) EEG, but in some cases interictal PET hypometabolism, ictal SPECT abnormalities, and MEG.
- invasive stereotactic-EEG (SEEG) and/or cortical electrical stimulation

#### Conditional data labelling for bias mitigation: topological studies

To evaluate and mitigate the expected publication bias favouring TLE, we collected Boolean information on whether a reported semiology originated from a study that preselected patients based on pre-specified brain regions. For example, a study stating ‘we looked at 100 patients with temporal lobe resections’ would have prior knowledge of the epileptogenic zone being the temporal lobe and would therefore be labelled as *epilepsy topology (ET)*. Stimulation studies were also considered a method of preselection as they assessed the semiology-generating potential of pre-specified cerebral regions.

All other articles were labelled *non-topological* e.g. articles reporting ‘we looked at 20 consecutive patients’ semiologies’ or ‘we evaluated 10 patients with ictal cough’ would be labelled *spontaneous semiology or non-topological*.

Data points were thus labelled as *topological* if they originated from either epilepsy topology or stimulation studies, and otherwise non-topological.

This method of data extraction enabled us to mitigate publication bias by filtering data points from studies that preselected patients based on prior knowledge of the seizure focus.

### Semiology taxonomy replacement

**Figure 2:**
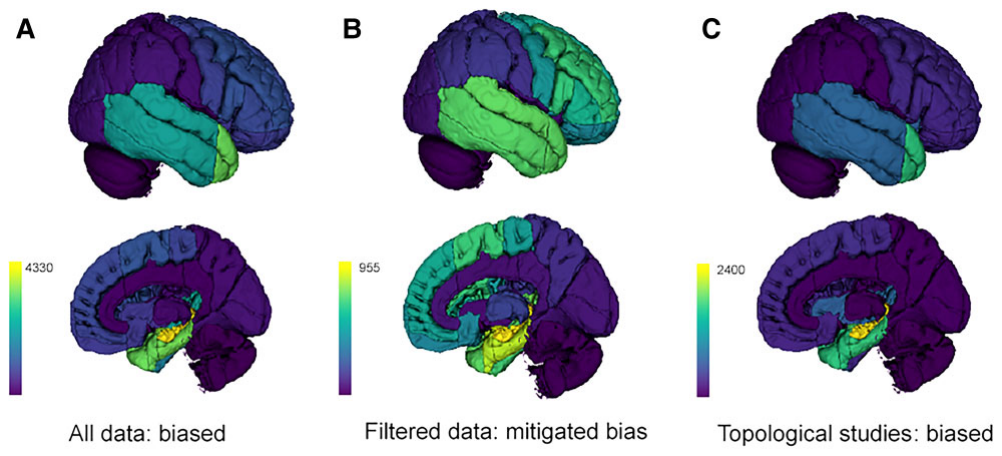
**Database overview and publication bias.** Semio2Brain Database overview. (**A–C**) Pseudo-glyph representations of integrated seizure semiology lateralizing and localizing values with data points (colour bars) obtained from querying the entire database (**A**); or querying non-topological studies only (**B**); or querying only data from topological studies where patients were preselected based on prior knowledge of their epileptogenic and seizure-onset zones (**C**). *Top* row: lateral views of the right hemisphere. Lower row: medial right hemispheres. These cortical heatmaps were obtained by querying the database for all semiologies. Colour bar represents number of data points.

A dictionary of regular expressions was devised as taxonomy replacement for seizure semiology categories, *SemioDict*. This searched both the semiologies as described exactly in the original article and the summary categories in *Semio2Brain* database (**2.1.1 Seizure semiology**), taking care to avoid mistakenly classifying negations and string similarities (e.g. myoclonic versus clonic and dystonic versus tonic). We included 35 broadly similar ictal semiological categories (Fig. 2D in purple) plus an additional postictal category and an asymptomatic category (absence of any reported semiology) for cortical stimulation studies. Descriptive definitions for each semiological category are summarized in **Table 1**. As this study was only interested in symptomatic ictal semiologies, we removed the postictal and asymptomatic categories before further analyses.

### Data processing and analysis

#### Querying Semio2Brain database

Although we queried the database for all 35 semiologies, we generated forest plots of localizing probabilities only for semiologies with at least 100 patients in both topological and non-topological data subsets so as to adequately capture the localizing distributions.

#### Risk of bias in *Semio2Brain* database

A Sankey diagram was used to visually assess patterns of publication bias and missing data points by year of publication, semiology, ground truths, topological priors, lobes, and age, with permutations in the order of layers (Supplementary Results).

#### Normalizing to number of patients

We normalized data points to set the unit of analysis to a single-patient semiology; such that the sum of all the localizing data points for all regions for a single semiology from a single patient would equal one. This has two effects: first, it favours semiologies that are more unifocal, by penalizing reports of semiologies that localize to multiple brain regions (inversely proportional to the number of brain regions to which the semiology of interest was localized). Second, it sets the sum of all data points for a semiology to be the number of patients in the literature who were reported to have had that semiology.

#### Localizing values: p(Localizing to region | Semiology)

Forest plots of semiological localizing values with 95% confidence intervals (CIs) were generated using 10 000 bootstrapped samples with replacement. We also assessed the intrinsic localizing value of each semiology relative to all other semiologies, by plotting the ORs for each semiology localizing to individual brain regions, with 95% bootstrapped CIs.

Three-dimensional representations of the distribution of localizing values from the corpus of semiological literature

Comparing non-topological versus all data for localizing values, we evaluated our best estimate for an unbiased prior distribution of localizations (EUD-Loc, Fig. 2B) for the entire database (all semiologies) and visualized this on 3D brain parcellations using the 3D-Slicer platform (https://www.slicer.org/).^26,27^ For details see Supplementary Methods.

### Statistical significance and implementation

All pre-processing, statistical analysis, and data visualizations were performed using python v3.6.10, and the packages: pandas v1.1.5, scipy v1.5.2, and plotly v4.9.0.^28–30^ Statistical significance was set at alpha = 0.05. The analytic code is available at https://github.com/thenineteen/Semiology-Visualisation-Tool/tree/kd-figures-v4

### Sensitivity analyses

#### Ground truths

As the ground truths were heterogenous, we explored the sensitivity of our probabilistic semiology localization values (forest plots) by using only the strongest ground truth that of postsurgical seizure-freedom, compared with using all three ground truths. Furthermore, we explored whether all data or filtered-data influenced this sensitivity analysis.

#### Age labels

We also performed sensitivity analysis to the age label in the database (Semio2Brain v.1.2.2) by excluding infants and children under 7 years, where this age label was available.

### Data availability

The open-access *Semio2Brain* Database is available at: https://github.com/thenineteen/Semio2Brain-Database. The *SemioDict* taxonomy is available in the resources folder at the repository: https://github.com/thenineteen/Semiology-Visualisation-Tool/tree/master/resources. The individual study screening table is available on request. The scripts to generate the forest plots and for statistical tests are available at https://github.com/thenineteen/Semiology-Visualisation-Tool/tree/kd-figures-v4/scripts/figures/figures.ipynb.

## Results

### Semio2Brain database v.1.2.2

A total of 11230 localizing and 2391 lateralizing data points were collected from 4643 patients across 309 included articles, all labelled for ground truths, topological priors, with localizing and/or lateralizing data points. Localizing data points grouped by topological priors are summarized in Fig. 2.

#### Evaluating for biases

The overall biased prior distribution of localizations (Fig. 2A) shows 66% temporal lobe localizations. As the majority of data points are from topological studies, the topological distribution of data points in Fig. 2C are even more biased towards the temporal lobes. Filtering out topological data to mitigate bias provides our best estimate for an unbiased distribution of localizations (EUD-Loc) as a prior for all seizure semiology in the literature, and is shown in Fig. 2B. This shows more balanced and widespread cortical localizations, mainly involving the temporal (44%) but also frontal lobes, based on ground truths of seizure-freedom, intracranial EEG and/or imaging and neurophysiological concordance.

A five-layer Sankey diagram (online only Supplementary Fig. 1) shows the localizing datapoint flows across the entire database: year of publication of study from which data was extracted (dark blue), ground truths (light blue), topological publication priors (orange), lobar localizations (yellow bars), and 35 ictal, 1 postictal, and 1 asymptomatic semiological categories (in purple). Lobes that have a majority of their data points from topological studies (orange links) in contrast to the minority of their data points from non-topological studies (yellow links) represent the topological publication bias favouring the temporal, occipital, and insular regions. Most of the database consists of epilepsy topology (ET) studies, and the majority of the ET output is to the temporal lobe and vice versa; therefore the majority of the publication bias is in favour of temporal lobe localizations. There was a maximum error rate of only 0.16% in Sankey data flow (online only Supplementary Fig. 1) due to missing data points, occurring at the level of the lobes.

The Sankey diagrams (online only Supplementary Figs. 1–3) highlight that the majority of the data points in the *Semio2Brain* database are from topological studies, and the majority of topological data points involve the temporal lobes (light orange links). Concurrently, the majority of temporal lobe data points are derived from topological studies. Other regions in which the majority of data points originate from topological studies are the occipital lobe and the insula (light orange topological inputs to these regions exceed their yellow non-topological inputs). These topological data points arise from studies that preselected patients based on knowledge that the occipital lobe or insula were the source of seizures. While representations of the database show a majority of non-topological data points also implicate the temporal lobes (Fig. 2B and online Supplementary Fig. 1), the insula does not feature in non-topological studies as prominently as it does in topological studies, suggesting that a high prior clinical suspicion is required to detect insular epilepsy.

### Seizure semiology localizing values

We queried the database for all *SemioDict* semiological categories. The definitions of the most commonly occurring semiologies are given in Table 1. These had more than 100 patients in both non-topological and topological subsets and were used for probabilistic and relative value (ORs) forest plots to ensure adequate numbers. Epigastric, olfactory, and somatosensory auras were the only three purely subjective ictal symptoms (as opposed to signs) amongst these 12 semiologies; autonomic auras constituted a mixture of symptoms and signs, and the other eight were ictal signs. These 12 semiologies made up the majority (65.5%) of normalized data points from non-topological studies (Table 1).

**Table 1:** Semiology descriptions and frequencies

Semiology category	Descriptions and examples	Percentage of non-topological data
Tonic	Stiff posturing of one or more limbs or torso	9.8%
Oral and manual automatisms	Upper limb automatisms, automotor (stereotyped distal limb movements), fiddling, pedal automatisms (excluding hypermotor or cycling), lip smacking, chewing, oro-alimentary, orofacial automatisms, ictal drinking, ictal swallowing	9.7%
Dialeptic-LOA-LOC	Blank stare, loss of awareness, unaware, loss of contact, psychomotor arrest, distant gaze, dreamy state, loss of consciousness (excluding generalized seizures) or dyscognitive states. Does not distinguish between partial or complete loss of consciousness.	8.3%
Epigastric	Abdominal rising sensation; e.g. butterfly sensation	6.1%
Vocalization—unintelligible noises	Grunting, mumbling, humming. Cf with ictal speech and dysphasia categories in Supplementary Materials (Supplementary Table 1)	5.5%
Autonomic	Autonomic symptoms or signs relating to any system, including respiratory, cardiovascular, genitourinary and gastrointestinal; e.g. hypopnoea, urinary urge, pilomotor or laryngeal constriction	4.7%
Olfactory	Any kind of ictal smell e.g. of burning	4.6%
Head version	Forced head deviation over the shoulder, extreme head turn	4.3%
Dystonic	Twisted posture or reported dystonia	3.4%
Other automatisms	Blinking, ictal cough, gelastic, dacrystic, ictal nose wiping and ictal face rubbing	3.1%
Mimetic automatisms	grimacing, raising of eyebrows, facial expressions e.g. fearful expression	3.1%
Somatosensory	Tingling or touch sensation	2.9%
All 23 other semiology categories	See Supplementary Table 1 for full list	34.5%

Twelve semiologies from the Semio2Brain database with their descriptions. Only those semiologies are shown where, after querying the database, the number of patients with localizing data for both the non-topological and topological subsets exceeded 100. The list is sorted in descending order of the number of patients with the semiology from the non-topological subset.

**Figure 3:**
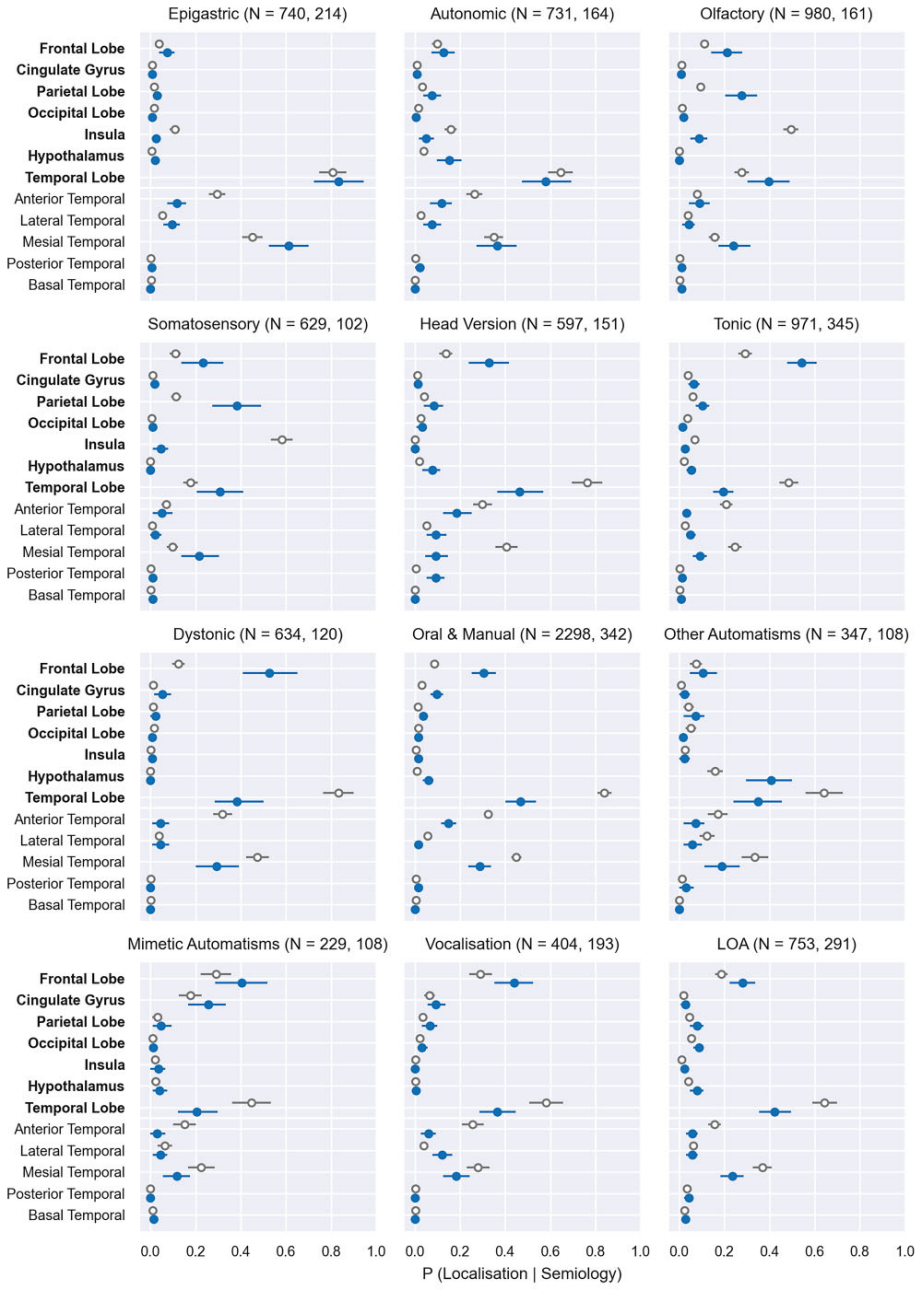
**Forest plots.** Seizure semiology localizing values for the 12 most commonly occurring semiologies: seven top-level brain regions are shown, and the temporal lobe is split into five subregions. The temporal lobe includes data points from its subregions as well undifferentiated localizations to the temporal lobe. Results from all data are in grey (empty circles) and spontaneous semiologies (non-topological studies) in blue (filled circles). Error bars represent 95% CI for 10 000 repeated bootstrapped samples. N, number of semiological data points (all data, non-topological subset). Data points are normalized to numbers of patients. LOA, loss of awareness. Oral and manual, orofacial automatisms and/or manual automotor signs.

The probabilistic landscape of the localizing values of these 12 semiologies are shown as forest plots in Fig. 3. The blue bars represent the probabilities of semiologies to localise to a region based on non-topological studies while in grey are the probabilities when including all-studies (both topological and non-topological). For semiologies clinically expected to localise to the temporal lobe such as epigastric auras, these two estimates were similar. Conversely, in semiologies such as **tonic** seizures that are clinically expected to localise to extratemporal regions,^15,31^ all data estimates are heavily biased towards the temporal lobe [48% 95% CI: (44%, 53%)], whereas non-topological estimates mitigate this by significantly reducing the temporal lobe estimate [20% (15%, 24%)], whereas revising up the estimate for **tonic** frontal lobe localization [all data 29% (26%, 32%) versus SS subset 54% (47%, 61%)].

If a patient had an **epigastric aura** as a manifestation of seizures, there was an 83% probability [95% CI: (72%, 94%)] that the seizure originated from the temporal lobe (specifically mesial temporal structures in 61% (52%, 71%), non-topological studies in Fig. 3). **Autonomic auras** indicated temporal lobe onset in 58% (47%, 67%) (mesial temporal in onset in 36% (27%, 44%)], with 13% (7%, 18%) having frontal and 15% (10%, 21%) hypothalamic sources. **Olfactory auras** were less specific, with 21% (15%, 28%) being frontal, 28% (20%, 35%) parietal, and 40% (31%, 49%) temporal in origin.

Undifferentiated **somatosensory auras** implicated three lobes [frontal 23% (15%, 32%), temporal 31% (21%, 42%), and parietal 38% (28%, 48%)]. **Head version** implicated temporal [46% (36%, 57%)] or frontal regions [33% (24%, 41%)], whereas **tonic** and **dystonic** seizures originated mainly from the frontal lobes [54% (47%, 61%) and 53% (40%, 66%), respectively].

**Oral and manual automatisms** were mainly temporal [47% (40%, 53%)] or frontal [31% (25%, 36%)] in origin. Other automatisms, of which more than half (62/108) were gelastic and dacrystic seizures (Table 1), implied an original source in the hypothalamus in 41% (30%, 50%), the temporal lobe in 35% (24%, 45%), or the frontal lobe in 11% (5%, 17%) of cases.

**Mimetic automatisms**, such as grimacing, mainly involved frontal [40% (29%, 52%)], cingulate [26% (18%, 33%)], and temporal lobes [20% (13%, 30%)]. **Non-sensical ictal vocalization**, such as grunting, was slightly more frontal in origin than temporal [44% (35%, 53%) versus 36% (28%, 45%)], whereas the reverse was true for **loss of awareness** (dialeptic seizures) [temporal 42% (36%, 49%) versus frontal 28% (23%, 34%)].

These results are broadly concordant with clinical expectations from studies of frontal and TLE seizure semiologies^13,15,31^ but are more nuanced with greater numbers of data points.

The insula featured mainly in topological studies due to publication bias (Fig. 2D), as indicated in the all data forest plots (Fig. 3 in grey) and only significant for the four subjective symptoms of epigastric (10%), autonomic (18%), olfactory (44%), and somatosensory auras (59%).

In these 12 seizure manifestations, the semiology that most significantly implicated the cingulate was mimetic automatisms 26% (18%, 33%) consistent with reports of anterior cingulate seizures demonstrating chapeau de gendarme (downturned mouth facial expressions),^32^ but the cingulate was also less frequently the source of seizures in oro-alimentary and manual automatisms 10% (7%, 13%), vocalization 9% (6%, 13%), tonic 7% (4%, 9%), dystonic 5% (2%, 9%), and dialeptic 3% (1%, 4%) semiologies, consistent with other reports.^33^

#### Probabilistic localizing values of seizure semiologies

##### Intrinsic localizing value of individual semiologies relative to all others

**Figure 4:**
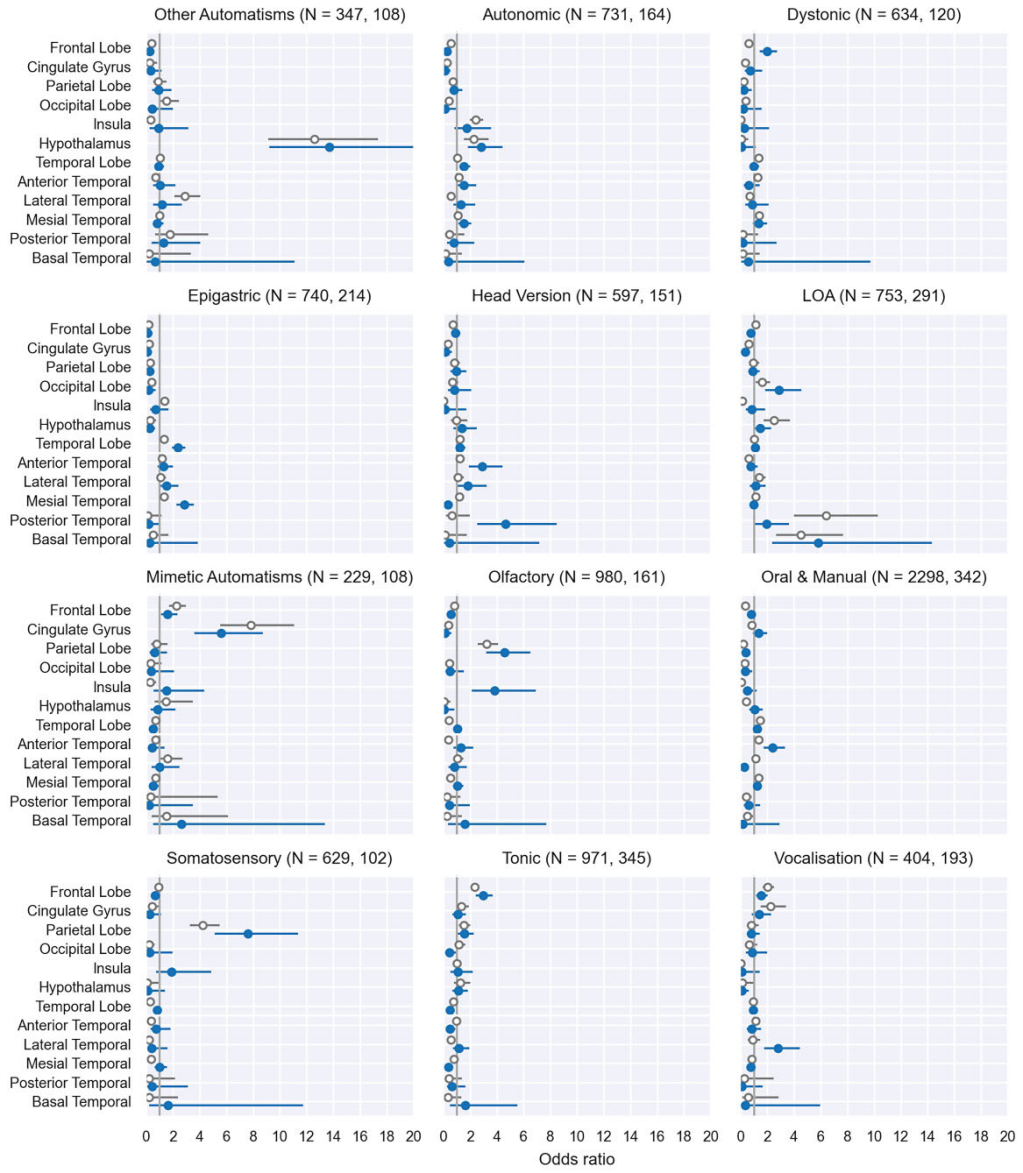
**Relative localizing values of semiologies:** Odds ratios of localizing value, given a semiology, for the 12 most commonly occurring semiologies in Semio2Brain database. These were calculated using two-by-two contingency tables from querying the entire Semio2Brain database for ictal semiologies. Blue (filled cirlces): spontaneous semiology (non-topological) data points. Grey (empty circles): all data.

The intrinsic localizing values of each semiology relatively to all others are shown in Fig. 4 as odds ratios, using internal semiological benchmarks. Semiologies that significantly deviate from the prior EUD-Loc (Fig. 2B)—compared with all other semiologies in the data—are shown in blue (non-topological) and grey (all data). The presence of **other automatisms** (Table 1) implicates the hypothalamus with an OR of at least nine [13.7, 95% CI: (9.2, 20.4)], whereas **autonomic features** involve the hypothalamus with OR 2.8 (1.8, 4.4). **Dystonic seizures** suggest frontal lobe onset with OR 2.0 (1.4, 2.7), and similarly **tonic seizures** intrinsically implicate the frontal lobes with OR 3.0 (2.4, 3.7). **Epigastric auras** implicate the temporal (specifically the mesial temporal) lobes with OR 2.4 (1.9, 2.9).

Although **head version** implicates the frontal and temporal lobes probabilistically [with probabilities 0.33 (0.24, 0.41) and 0.46 (0.36, 0.57) respectively, Fig. 3], it does not add significant value relative to our prior expectation that the source of seizures is likely to be from the frontal [OR 0.9 (0.7, 1.2)] or temporal lobes [OR 1.21 (0.9, 1.6)]. That is, the knowledge that a patient has head version (ORs in Fig. 4) does not significantly revise our expectation compared with before knowing any specific semiology (EUD-Loc Fig 2B). This can be attributed to temporal and frontal lobe epilepsies being the two most common localization-related epilepsies. Head version does however seem to carry intrinsic value for the posterior and anterior temporal subregions (Fig. 4).

**Loss of awareness,** whether in isolation or accompanying other semiologies, implicates the occipital lobe with OR 2.9 (1.8, 4.6). Loss of awareness also has intrinsic value in implicating the posterior and basal temporal subregions [OR 2.0 (1.0, 3.6), OR 5.8 (2.4, 14.3), respectively; Fig. 4).

**Mimetic automatisms** such as grimacing localizes to the cingulate gyrus with OR 5.6 (3.6, 8.7), while **Olfactory auras** implicate both parietal [OR 4.6 (3.2, 6.5)] and insular regions [OR 3.8 (2.1, 6.9)].

**Oral and manual automatisms,** such as lip smacking and chewing movements, do not significantly implicate the temporal lobes more than the prior EUD-Loc, but do show a propensity towards the anterior temporal subregion [OR: 2.4 (1.7, 3.3)], probably due to the successful and commonly performed anterior temporal resections in individuals with TLE.

Somatosensory auras localize to the primary somatosensory cortex within the parietal lobes, OR 7.6 (5.1, 11.3), showing that its presence as an early or prominent ictal symptom should significantly steer the clinician towards the parietal lobe. The intrinsic localizing value of somatosensory symptoms to the insula is statistically non-significant [OR: 1.9 (0.7, 4.9)].

**Vocalizations** (unintelligible noises) intrinsically localize to the frontal lobe with an OR 1.5 (1.2, 2.0) and the lateral temporal subregions [OR 2.8 (1.8, 4.5]).

### Sensitivity analyses

Supplementary Figure 4 shows the probabilistic localizing values when using only the ground truth of postsurgical seizure-freedom. This forest plot is similar to that of using all ground truths (Fig. 3), as can be appreciated in when overlaying the results from Fig. 3 with that of Supplementary Fig. 4.

Supplementary Figure 5 compares all data (topological and non-topological) results from Fig. 3 with all data results of the single ground truth of seizure-freedom from Supplementary Fig. 4.

Supplementary Figure 6 directly compares the results from topological filtered-data from Fig. 3 with filtered-data from the single ground truth of seizure-freedom in Supplementary Fig. 4.

Supplementary Figures 5 and 6 show robust results and overlap in CIs, with the exception of a lack of hypothalamic data points in seizure-freedom all data (Supplementary Fig. 5: ‘autonomic’, ‘other automatisms’ and ‘LOA’) and a lack of hypothalamic data points in seizure-freedom filtered-data (Supplementary Fig. 6: including the three aforementioned semiologies, as well as ‘Tonic’, ‘Head Version’, ‘Oral and Manual Automotor’).

The probabilistic localizing values from all ground truths when excluding data from children aged younger than 7 years was also similar to that of the probabilistic forest plot of all ages as shown in Fig. 3 (Supplementary Fig. 7).

Therefore, in summary, the probabilistic localizing values obtained using all ground truths (Fig. 3), were robust to sensitivity analysis using only the ground truth of postsurgical seizure-freedom, for all regions, and semiologies; with the exception of hypothalamic data points (Supplementary Results). The probabilistic localizing values obtained using all ground truths (Fig. 3) was also robust to excluding patients aged younger than 7 years of age.

## Discussion

Epilepsy affects 50 million people worldwide, and one-third continue to have frequent seizures despite medications. Surgery can be curative if a seizure focus is identified,^34^ but less than half of resections result in complete seizure-freedom.^4,35^ Epileptic symptoms and signs help to localize the seizure focus in the evaluation of patients with drug-resistant focal epilepsy for curative surgery, but few clinical experts can interpret these seizure manifestations^6^ and the art is somewhat subjective. We created the largest database linking ictal symptoms and signs to lobar and sub-lobar localizations (*Semio2Brain* v.1.2.2, 2021, doi:10.5281/zenodo.4473240). *Semio2Brain* is a fully open-source and data-driven database obtained from a PRISMA-guided systematic review of the corpus of seizure semiology publications with more than 11 000 localizing data points from 4643 patients across 309 peer-reviewed publications. In this study, we described the objective clinical values of seizure semiology in terms of lobar localization, by using ground-truthed data and applying a Bayesian data filter whereby probabilities of lobar localization given a semiology were not mixed with studies that preselected patients based on prior knowledge of their epileptogenic foci. We showed that Bayesian filtering (non-topological studies) more accurately represented clinical expectations but also provided more nuanced information by quantifying the localizing distributions of different semiologies. Results were robust to sensitivity analyses by known age labels and postsurgical seizure-freedom ground truth.

### *Semio2Brain* database and publication bias

The localizing probabilities of semiologies can be obtained from the literature to capture brain areas that determine observed ictal signs and experienced seizure symptoms. The novelty of our approach was threefold: first, we curated data from a systematic review of 1194 screened articles resulting in full-text data extraction from 309 publications across many different centres over seven decades (earliest publication included in *Semio2Brain* is from 1954).

Second, we mitigated publication bias through conditional data labelling of studies that described patients’ semiology based on prior knowledge of their seizure foci (topological studies, such as a case series of TLE or cortical stimulation studies). The cortical heatmap summary of all topological studies (Fig. 2C) and the Sankey diagram (Fig. 2D, interactive online) clearly demonstrate temporal lobe bias, whereby 81.7% of temporal lobe data points arise from topological studies, and 75% of topological data points localise to the temporal lobe. This temporal lobe bias in the literature is expected, as TLE occurs both most commonly and has the best surgical outcomes.^34,36–38^

Third, we mitigated bias by filtering results using the topological labels in the *Semio2Brain* database, to approximate the conditional probability of localizing to any particular brain region given a specific semiology. By comparing unfiltered (all data) results with filtered (non-topological data only) localizing data points in forest plots, we showed that data filtering more accurately captured extratemporal localizations, mitigating frequentist bias which would otherwise implicate the temporal lobe as the source of seizures in eight of 12 of the most commonly occurring semiologies. These eight semiologies in which the filter (non-topological studies) significantly reduced the probability of localization to the temporal lobe were: head version, tonic, dystonic, orofacial, and manual automatisms, other automatisms including gelastic seizures, mimetic, unintelligible vocalizations, and episodes of loss of awareness (dialeptic) (Fig. 3).

### Localizing probabilities

Even if cortical seizures are stable and reproducible from neurophysiological and semiological perspectives in individuals,^39^ marked variations can exist between patients. In addition, dense neural connections result in rapid seizure propagation within and between cerebral hemispheres,^9^ leading to variable semiology even within an individual, limiting the value of univariate methods in localizing semiology. Therefore, we propose that the manifestations of cortical stimulations and the semiology of a given brain region are best considered non-injective surjective mappings involving network nodes. That is, seizures arising in any part of an isolated early spread network will manifest in a stereotyped manner with a small variance, but any specific semiology can arise from disparate network nodes with a larger variance. We modelled this latter case as a conditional probability of localization given a semiology and showed that the set of non-topological studies more accurately represent this conditional probability than topological studies. As *Semio2Brain* is the largest ictal phenotype database with over 11 000 localizing data points for semiologies, we were able to capture these variances in semiological localizing values and display results as forest plots at the lobar (and sub-lobar) levels.

Our best estimate for the unbiased prior distribution of localizations from the literature (EUD-Loc, Fig. 2B) used mixed ground truths of postoperative seizure-freedom, imaging and neurophysiological concordance, and invasive EEG. As EUD-Loc was derived from non-topological studies, it is the closest attempt thus far at accurately capturing the distribution of epileptogenic anomalies in the brain from the literature at the resolution of seven brain regions (temporal, frontal, parietal, and occipital lobes; insula, cingulate, and hypothalamus). The EUD-Loc and semiology-specific probabilistic localizing values derived from the database are consistent with observations that distributed epileptogenic networks are often involved during seizures and can be used as prior probabilities of epileptogenic abnormalities in applications of network theory to focal epilepsy.^22^ Our forest plots provide the probabilistic localizing values for major network nodes that may be involved in the production of the most common semiologies, capturing the combined concepts of seizure onset, symptomatogenic, lesional, irritative, and epileptogenic zones that constitute our underlying ground truths.^3,13,40^

For example, although frontal, temporal, and hypothalamic regions are known to be involved in the production of gelastic and dacrystic seizures, the probabilities of their involvement have not been adequately quantified.^14^ Our filtered forest plots quantified these probabilities (Fig. 3). As a further example, ictal unintelligible vocalizations mainly involved distributed frontal and temporal networks (filtered Fig. 3) in line with previous studies investigating the distributed networks of lexical retrieval.^41^ We also found that these non-sensical ictal vocalizations (such as grunting), whether in isolation or as co-occurring semiologies, were of frontal or temporal origin in most cases but could not definitively differentiate between the two lobes [44% (35%, 53%) versus 36% (28%, 45%), respectively). Complementary to this finding, a previous study of 102 patients with ictal vocalization showed high sensitivity (91%) and specificity (70%) for detecting temporal lobe seizures when vocalizations co-occurred with automatisms but not alone.^42^

Furthermore, in semiologies with established network models, such as functional-MRI activation changes in the default mode network associated with impairments in consciousness or dialeptic episodes,^14^ our forest plots quantified the diverse localizations to all seven regions: temporal 42% (36%, 49%), frontal 28% (23%, 34%), occipital 9% (6%, 11%), parietal 8% (5%, 11%), hypothalamus 8% (5%, 10%), and cingulate and insula both under 5% (1%, 4%). These results are consistent with other studies on the value of altered consciousness in focal seizures, suggesting they may originate mainly from the temporal lobe (but unquantified)^43^ or multiple brain regions including 35% from temporal, 16% from frontal and 5% from parieto-occipital regions.^44^

The *Semio2Brain* open-source database and derived results have the potential to be complementary to lesion-deficit mappings and can serve as the basis of future phenotypic imaging, whereby ictal symptoms and signs are probabilistically mapped to cortical epileptogenicity.

### Relative localizing values using odds ratios

While many studies have evaluated the localizing values of semiologies,^9,13,15^ fewer have explored its relative value compared with other investigative tools such as EEG, PET, or MRI,^19^ or quantified the additional value semiology provides alongside other modalities such as the combination of semiology and the MRI finding of hippocampal sclerosis for the diagnosis of TLE.^38^ No study has evaluated the intrinsic relative value of any one semiology over all other ictal manifestations, mainly due to the absence of sufficient data. This was made possible through our collection of 4643 patients’ data. Combining thousands of semiological localizing data points from the non-topological data subset enabled us to estimate the relative localizing values of each semiology compared with all others. In effect, the intrinsic values of semiologies presented in this study (as ORs) approximate the EUD-Loc as a prior benchmark and evaluate to what degree a particular semiology’s localizing odds diverge from this.

To illustrate this, we could consider the probabilistic transformation of the EUD-Loc (shown as a frequency heatmap in Fig. 2B and a Sankey diagram in Fig. 2D) as a good clinical estimate for the source of seizures in patients with focal epilepsy prior to having any clinical information or investigation results, mainly favouring temporal (44%) and frontal lobe (31%) epilepsies. Subsequently, knowledge about the presence of any particular semiology e.g. epigastric auras, will then update our prediction for considering the temporal lobe as the source of seizures with an OR of 2.4 (1.9, 2.9) [specifically the mesial temporal OR 2.8 (2.3, 2.9), Fig. 4). **Epigastric auras** localise to the temporal lobe with 83% probability [95% CI (72%, 94%)] (Fig. 3), but this does not take into account that at baseline there is a higher likelihood that the temporal lobe is involved than any other brain region (EUD-Loc Fig. 2B).

In EUD-Loc (non-topological) there is ∼44% probability of the temporal lobe being the source of seizures before knowing the semiology, this is in contrast to using combined topological and non-topological data points which would return a prior estimate for TLE of over 66% (all data Fig. 2A).

Therefore, ORs with 95% confidence intervals not overlapping 1 for any given semiology in Fig. 4 signify value-added localizing information over and above the baseline frequencies, and these semiologies and their localizations help to narrow the likely seizure sources.

Although we have shown the relative localizing values of the 12 most commonly occurring semiologies, the ORs were calculated using all semiologies that occur in *Semio2Brain database*, including the less frequently occurring semiologies (Supplementary Table 1).

### Semio2Brain database: future uses

#### Mapping seizure phenotypes to cortical epileptogenicity

The *Semio2Brain* database can serve as the foundation for phenotypic imaging, whereby ictal symptoms and signs are probabilistically mapped to cortical epileptogenicity, which if clinically validated could help objectively localise seizure foci in the evaluation of individuals with fDRE.

#### Lateralizing values

Semio2Brain contains lateralizing information relative to semiology and language dominance that can be used to determine the lateralizing values of semiologies as we have done for their localizing values.

#### Comparisons by ground truths

The data and analyses can be filtered by ground truths to compare the values and effects of the epileptogenic, symptomatogenic, irritative, lesional, and seizure-onset zones (Supplementary Figs. 4 and 5).^3,13,40^

#### Generative models of seizure semiology

Seizures with similar semiologies are thought to involve abnormal paroxysmal neuronal discharges that originate and propagate within concordant brain networks. We used frequency analysis of semiologies localizing to brain regions to describe the probabilistic and intrinsic relative values of seizure semiology. Because the *Semio2Brain* database captures the partial set of semiologies from the literature when chronology was unspecified, its topological studies could be used to derive the reverse conditional probabilities of brain regions’ abilities to generate ictal symptoms and signs as a proxy for rapid seizure propagation to other regions within its network.

Although similar methods have shown topological organization of brain regions and semiology, such as in 54 patients with hierarchical clustering of 24 frontal lobe regions and 31 ictal signs,^9^ this has not been directly compared with structural or functional connectivity correlations between the same cortical regions to investigate the degree to which seizure manifestations may arise from underlying brain connectomes. The thousands of patients in *Semio2Brain* enable this comparison. A future model built on the topological subset could be the basis of a generative model of ictal phenotypes for incorporation into Bayesian virtual epileptic brain models^45^ and could also be used to obtain a semiological connectivity matrix for comparison with structural, functional, and electrographically derived dynamic connectivity measures.^46^ Such analyses could ascertain the degree to which semiology and connectivity measures may be correlated and elucidate the extent to which seizure manifestations are single-node or network driven.^8^ This may lead to integration of semiological sequence predictions with propagation zone predictions for any given epileptogenic zone in personalized virtual brain network models.^47^

### Limitations

There are inherent limitations in using descriptions of semiology^8^ and descriptions of regions of interest to develop probabilistic localizing models. Errors can be introduced at multiple stages including publication bias, data collection, mapping to both hierarchical regions and semiologies, and during normalization (Supplementary Results).

Imaging and neurophysiological concordance may not be as strong a ground truth as postoperative seizure-freedom, and the seizure-onset zone determined by SEEG may be part of a larger early spread network still downstream to the initial seizure focus,^7^ adding noise to the localizing values. For example, posterior cingulate epilepsy can have electroclinical findings that mimic a temporal lobe origin,^48^ reducing the number of cingulate data points from the concordance ground truth.

When the semiological chronology was specified in the literature, only the initial semiology was collected. However, semiologies reported without specified chronology were collected (regardless of their ictal time of onset). Therefore, due to semiological reporting bias, the collected semiologies in this study are not all the earliest semiology, but rather a mix of both initial (or co-occuring) and other semiologies, adding further noise to the findings. Because no chronological evolution is available in the database, it was impossible to include seizure evolution information in our EUD-Locs, likely resulting in a relatively poor estimate of EUD-Locs. Temporal evolution data was frequently not given in the literature and this is a limitation of the data.

Nevertheless, this may make the results from this study more clinically applicable for predicting localization, as semiologies reported in clinic are not always chronologically accurate; for example, some early experiential auras may not be recollected at all and if they are recollected, only the most prominent aura (rather than the initial aura) may be reported.^49^

Semiology varies by age, reflecting brain maturation and shifts in propagating networks, so children’s semiologies differ from adults.^50–52^ In this study, we looked at all ages and the known adult subgroup only. In future, we hope to evaluate paediatric data separately.

An inevitable caution is that the symptomatogenic zone, that generates the observed semiology, may be distant from the seizure-onset zone. Thus, semiological analysis may only infer the likely localization of the site of seizure onset.

See also Supplementary Limitations.

## Conclusions

We present the largest data-driven and open-access database, *Semio2Brain*, for early seizure semiology consisting of 11 230 localizing data points from 4643 patients across 309 publications, with ground truths for localizations. We investigated and mitigated publication bias using topological data filtering. As a specific semiology can arise from disparate brain nodes, we modelled this as a conditional probability of localization given a semiology and showed that the set of non-topological studies in *Semio2Brain database* more accurately represented this than topological studies. As *Semio2Brain* is the largest ictal phenotype database, we were able to capture these variances in semiological localizing values and display results as forest plots at the lobar (and sub-lobar) levels.

We therefore paint the probabilistic localizing landscape of the 12 most commonly occurring semiologies, and their intrinsic localizing values relative to any other semiology. We also propose other potential uses for the database including a generative model of seizure semiology.

## Supplementary Material

fcac130_Supplementary_Data

## Abbreviations

EUD-Loc =: Estimate of Unbiased Distribution of Localizations
CS =: cortical stimulation
ET =: epilepsy topology
fDRE =: focal drug-resistant epilepsy
FL =: frontal lobe
LOA =: loss of awareness
LOC =: loss of consciousness
SPECT =: single-photon emission computed tomography
TL =: temporal lobe
