# Supplementary material for: Probabilistic landscape of seizure semiology localizing values
Source: Brain Commun. 2022 May 19;4(3):fcac130. doi: 10.1093/braincomms/fcac130 (PMC9156627; doi:10.1093/braincomms/fcac130)
Supplement: fcac130_Supplementary_Data [file fcac130_supplementary_data.zip › Supplementary_Materials.docx]

Supplementary Materials

Probabilistic Landscape of Seizure Semiology Localising Values

Contents

[**1.** **Abbreviations** 3](#_Toc73612903)

[**2.** **Methods** 3](#_Toc73612904)

[2.1. Search Terms 3](#_Toc73612905)

[2.2. Inclusion criteria 3](#_Toc73612906)

[2.3. Exclusion criteria 3](#_Toc73612907)

[2.4. Semio2Brain Preprocessing 3](#_Toc73612908)

[2.4.1. Interlobar junctions localising datapoint redistributions 4](#_Toc73612909)

[2.5. SemioDict Terminology 6](#_Toc73612910)

[2.5.1. Supplementary Table 1: Descriptions of All Categorised Semiologies in SemioDict 6](#_Toc73612911)

[**3.** **Supplementary Results** 11](#_Toc73612912)

[3.1. Normalised vs not normalised data (all-data) 11](#_Toc73612913)

[3.2. Visual Bias Assessment: Sankey Permutations 11](#_Toc73612914)

# **Abbreviations**

TL = Temporal Lobe

FL = Frontal Lobe

PL = Parietal Lobe

OL = Occipital Lobe

LOA = Loss of awareness

LOC = Loss of consciousness

# **Supplementary Methods**

## Search Terms

As seizure semiology with localising ground-truths data from any single centre is limited, we used a PubMed search of the primary literature as the source for our Semio2Brain datapoints. The following search terms were used in January 2019 with filters for human studies, abstract and full-text availability, in English, French, German, Italian or Spanish:

((focal OR partial) AND (epilep* OR seizure OR ictal OR interictal OR aura) AND (local* OR lateral* OR map*) AND (semiolog* OR signs OR symptoms OR phenotype OR features OR characteristics)) AND ((zone OR source OR focus OR foci)) OR ((epilep* OR seizure OR ictal OR interictal) AND semiolog*)

One consultant neurologist (AAM) and one post-doc (GR) collected all the data, alternating between one collecting and the other checking. In the event of disputes, another consultant neurologist served as a tie-breaker (JD).

## Inclusion criteria

Publications must have been regarding patients with focal drug-resistant epilepsy, with available details on seizure semiology and either descriptions or the actual imaging of the localising and/or lateralising regions. Data must have clearly met one or more ground truths for inclusion, as stated above. If semiology sequence was not determined, all the reported semiologies were collected, otherwise only the initial semiologies were collected. There was no age limit. Studies in English, French, Spanish, Italian or German were included, but the abstract must have been in English.

## Exclusion criteria

Exclusion criteria were generalised epilepsy, kappa <0.4 for semiological inter-observer agreements (where provided), broad non-specific localising terms (e.g., “extra-temporal”), unmet ground truth criteria, or significant metabolic derangement in the context of multi-organ failure or critical care. Additionally, if a patient had had a surgical resection, they were excluded from meeting other ground truths if they were not seizure-free (Engel I) irrespective of the presence of multimodal concordance or SEEG.

## Semio2Brain Preprocessing

As there were only 10 cerebellar datapoints from a total of 11230, we omitted the cerebellum as a region altogether; none of the reported semiologies were affected.

*Semio2Brain’s* brain regions have shorthand labels for interlobar junctions, whose data-points were redistributed across the respective lobes as below during preprocessing.

### Interlobar junctions localising datapoint redistributions

Interlobar junctions datapoints from the Semio2Brain database were redistributed as such:

Fronto-temporal:

• Frontal

• Insula

• Lateral Temporal

• TL

Temporo-occipital:

• Lateral Temporal

• TL

• OL

Temporo-parietal:

• Lateral temporal

• TL

• PL

Fronto-temporo-parietal:

• Insula

• Lateral temporal

• TL

• FL

• PL

Temporo-parieto-occipital Junction:

• Lateral temporal

• TL

• PL

• OL

Parieto-occipital:

• PL

• OL

Fronto-parietal:

• FL

• PL

Perisylvian:

• Insula

• Lateral temporal

• TL

• FL

• PL

Subcallosal cortex:

• Anterior cingulate

• Cingulate

Note that only the temporal subregions were used for the redistribution, as the majority of the datapoints pertained to the temporal lobe and its subregions, and these were the only subregions we could show in forest plots that had sufficient numbers of datapoints for all semiologies.

## SemioDict Terminology

### Supplementary Table 1: Descriptions of All Categorised Semiologies in SemioDict

| **Category** | **Subset Examples** | **Comments** |
| --- | --- | --- |
| **Aphasia** | mute, speech arrest, aphemia anarthria | does not include postictal aphasia |
| **Astatic** | drop attack | does not differentiate between tonic or atonic astatic |
| **Asymmetric Tonic** | fencing, figure of 4 | laterality is with respect to raised/extended arm |
| **Atonic** | flaccid, jelly, head drop |  |
| **Auditory** | hearing sounds, auditory hallucination |  |
| **Automatism - Automotor Manual and Oral** | upper limb automotor, fiddling, pedal, automatisms, lip smacking, chewing, oroalimentary, orofacial automatisms,  ictal drinking, ictal swallow | pedal automatisms exclude hypermotor pedalling or cycling leg movements |
| **Automatisms - Other** | blink, ictal cough, gelastic, dacrystic  spitting, ictal nose wiping, ictal face rub |  |
| **Autonomic** | cardiovascular e.g. ictal bradycardia,  respiratory e.g. hypopnoea  gastrointestinal: e.g. nausea | also includes urinary e.g., urinary urge, pilomotor and laryngeal constriction |
| **Clonic** | repetitive or rhythmical jerks |  |
| **Complex Behavioural** | behavioural change, fearful behaviour, wandering, awakening or arousal, compulsive checking | cf. fearful behaviour with fearful expression under mimetic |
| **Dialeptic-LOA-LOC** | blank, unaware, loss of contact stare, distant gaze, dreamy state, dyscognitive, not with it  blackout, psychomotor arrest, loss of consciousness, unconscious | does not distinguish between partial and complete loss of consciousness |
| **Dysphasia - Ictal Speech** | difficulty speaking or incoherent speech, expressive dysphasia, incomprehensible but formed words, ictal speech, palilalia, coprolalia | cf. vocalisation (nonsensical sounds). Excludes postictal dysphasia. NB potential overlap with vocalisation and ictal speech |
| **Dystonic** | twisted posture |  |
| **Epigastric Aura** | abdominal aura, butterfly sensation, rising sensation |  |
| **Eye Movements** | nystagmus (fast phase direction), ocular flutter, complex ocular movements, gaze deviation and versive eye movements |  |
| **Fear-Anxiety** | sense of impending doom, fear, anxiety, negative emotion | cf. psychic category |
| **Gustatory** | taste aura |  |
| **Head or Body Turn** | head turn, gyroscopic or body turn | Cf. head version which is forced head turning |
| **Head Version** | forced head deviation over shoulder, extreme head turn | cf. head turn which is unforced with milder head turning |
| **Hypermotor** | large proximal limb or axial movements, hyperkinetic, head banging, pedaling, kicking, pelvic thrust |  |
| **Hypomotor** |  | note this is a paediatric only semiology, used when it is  difficult to differentiate between behavioural arrest or dialeptic semiologies |
| **Ictal Limb Paresis** | ictal hemiparesis, ictal hemiplegia |  |
| **Ictal Pout** | chapeau |  |
| **Mimetic Automatisms** | grimace, raising of eyebrows, mimetic, facial expression, fearful expression |  |
| **Myoclonic** | Jerk |  |
| **No Semiology - Only Stimulation Studies** | when stimulation has no semiology,  clinically silent electrographic seizure | a report of no subjective or objective semiology during cortical stimulation |
| **Non-Specific Aura** | vague, unspecified aura lightheaded, dizzy, indefinable feeling, cephalic sensation |  |
| **Olfactory** | smell |  |
| **Postictal Aggressive** |  | excludes ictal |
| **Postictal Aphasia** |  | excludes ictal |
| **Postictal Cough** |  | excludes ictal |
| **Postictal Dysphasia** | postictal dysphasia and paraphasia | excludes ictal |
| **Postictal Hemiparesis or Hemiplegia** | Todd's paresis or paralysis | excludes ictal |
| **Postictal Nose-wiping** |  | excludes ictal |
| **Postictal Oral or Manual Automatisms** | postictal oral automatisms, postictal automotor, postictal drinking | excludes ictal |
| **Psychic** | experiential, affective, erotic, déjà vu, deja vecu, jamais vu, derealisation, depersonalisation | excludes fear/anxiety which has been categorised separately |
| **Somatosensory** | tingling, touch sensation |  |
| **Spasms** | infantile spasm and epileptic spasm | usually used in paediatric cases, however, Semio2Brain data from publications also included adults |
| **Tonic** | stiff, tonic posturing | excludes tonic and clonic |
| **Tonic-Clonic** | tonic and clonic together | excludes tonic only and excludes secondarily generalised, therefore few cases |
| **Vestibular** | vertigo, spinning sensation |  |
| **Visual** | formed visual hallucinations e.g., people or objects, movement of objects (not vestibular), phosphene, macropsia, micropsia, metamorphopsia |  |
| **Vocalisation - Unintelligible Noises** | grunt, mumble, hum | cf. ictal speech and dysphasia |

Supplementary Table 1: Full list of SemioDict semiology categories and descriptions

## 3D-brain glyph representations of localising values of semiology

The 3D cortical heatmaps in the main manuscript Fig. 2 were created by querying all *Semio2Brain* datapoints with left laterality (where the semiology allowed lateralisation e.g., tonic) and mapping the descriptive brain regions to a geodesic information flow parcellation^1^ and then displayed on a summary brain in Montréal neurological institute (MNI152) space with left hemisphere dominance using 3D-Slicer v4.11.0 (<https://www.slicer.org/>)^2^, to give a pseudo-glyph representation of the biased and unbiased data. The code to perform this is available at <https://github.com/thenineteen/Semiology-Visualisation-Tool>.

# **Supplementary Results**

## Normalised vs not normalised data (all-data)

| **Brain Region** | **Not Normalised** | **Normalised** |
| --- | --- | --- |
| Temporal Lobe | 0.589 | 0.626 |
| Frontal Lobe | 0.158 | 0.137 |
| Cingulate | 0.034 | 0.022 |
| Parietal Lobe | 0.046 | 0.038 |
| Occipital Lobe | 0.032 | 0.033 |
| Insula | 0.126 | 0.128 |
| Hypothalamus | 0.015 | 0.016 |

Supplementary Table 2: the effect of normalisation on unfiltered all-data lobar localisations in Semio2Brain database.

## Visual Bias Assessment: Sankey Permutations

**Supplementary Figure 1** attached as a supplementary file is an interactive Sankey diagram. This shows year of publication, ground truths, topological priors, lobes, and semiologies. Publication biases from topological studies are highlighted with pink links favouring the temporal and occipital lobes as well as the insulae. The rest of the datapoint flows appear to have sufficient variance of sources and destinations.

SF: seizure free. sEEG: stereotactic EEG. ET: epilepsy topology. CS: cortical stimulation. SS: non-topological spontaneous semiology. TL: temporal lobe. PL: parietal lobe. OL: occipital lobe. FL: frontal lobe. LOC: loss of consciousness.

**Supplementary Figure 2** is attached as a supplementary file is an interactive Sankey diagram, showing the biases again in pink, but this time with the temporal lobe split into mesial, anterior, lateral, and basal regions. Biases are shown to be present for all of these subregions. Additionally, there is a labelled age layer showing the majority of datapoints in the database version we used had no documented age.

**Supplementary Figure 3** looks at the flows between topological priors and semiological categories (layers three and four). It does not show significant biases other than the fact that the majority of datapoints generally come from topological studies. Layers four and five show flows from semiologies to lobes which are also sufficiently varied. The last two layers show that the majority of insular datapoints originate from individuals over 7 years old, while the majority of interlobar junction (“other”) datapoints originate from children under 7 years.

## Sensitivity Analysis Results

### Ground Truth: Postsurgical Seizure-Freedom Only


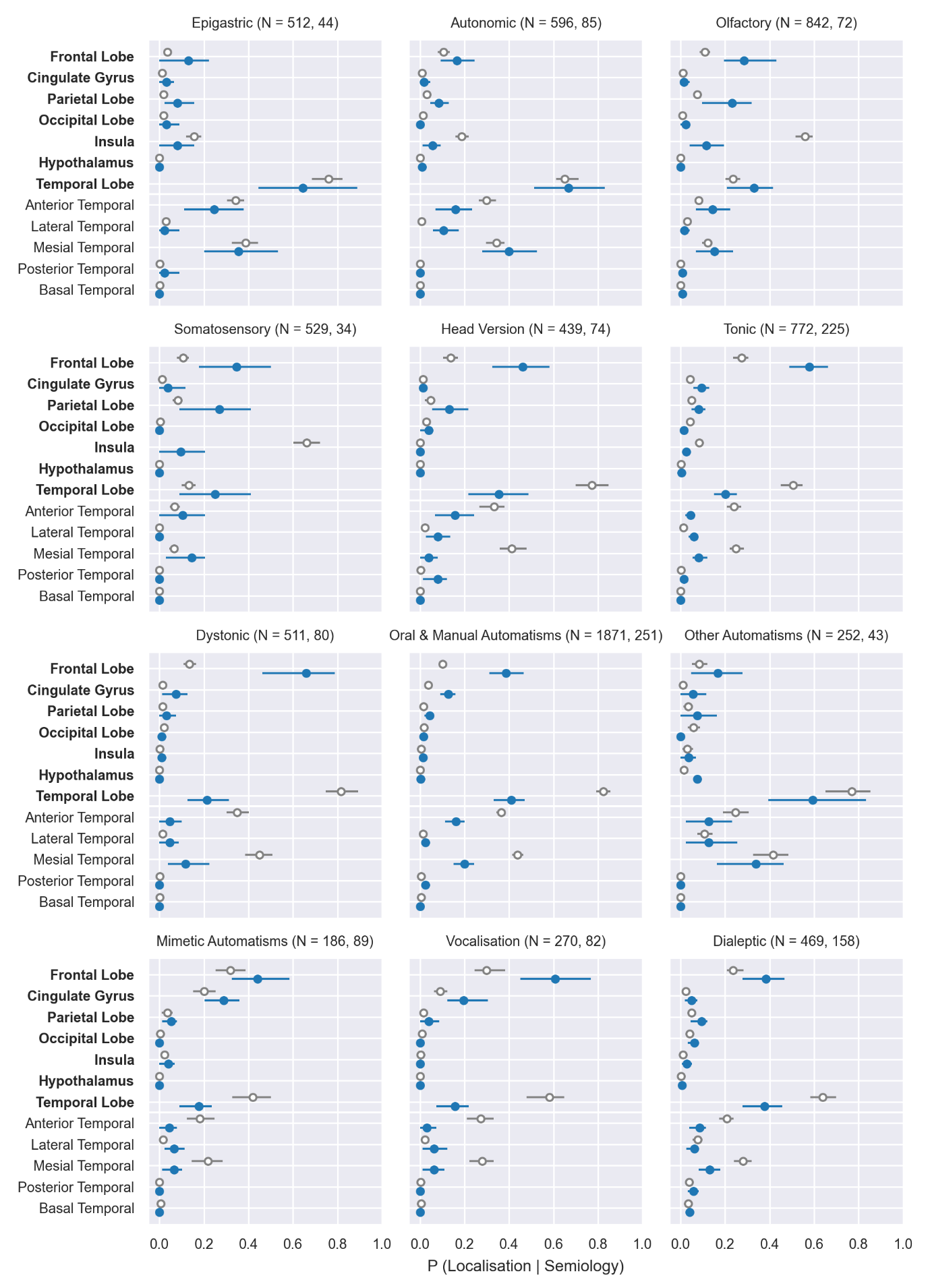


**Supplementary Figure 4 Seizure-freedom ground-truth only: localising values for the 12 most commonly occurring semiologies.** This Figure is the sensitivity analysis counterpart to main manuscript figure 3, using only the ground-truth of postsurgical seizure-freedom. Empty grey circles are all-data, filled blue circles are filtered-data (non-topological only).


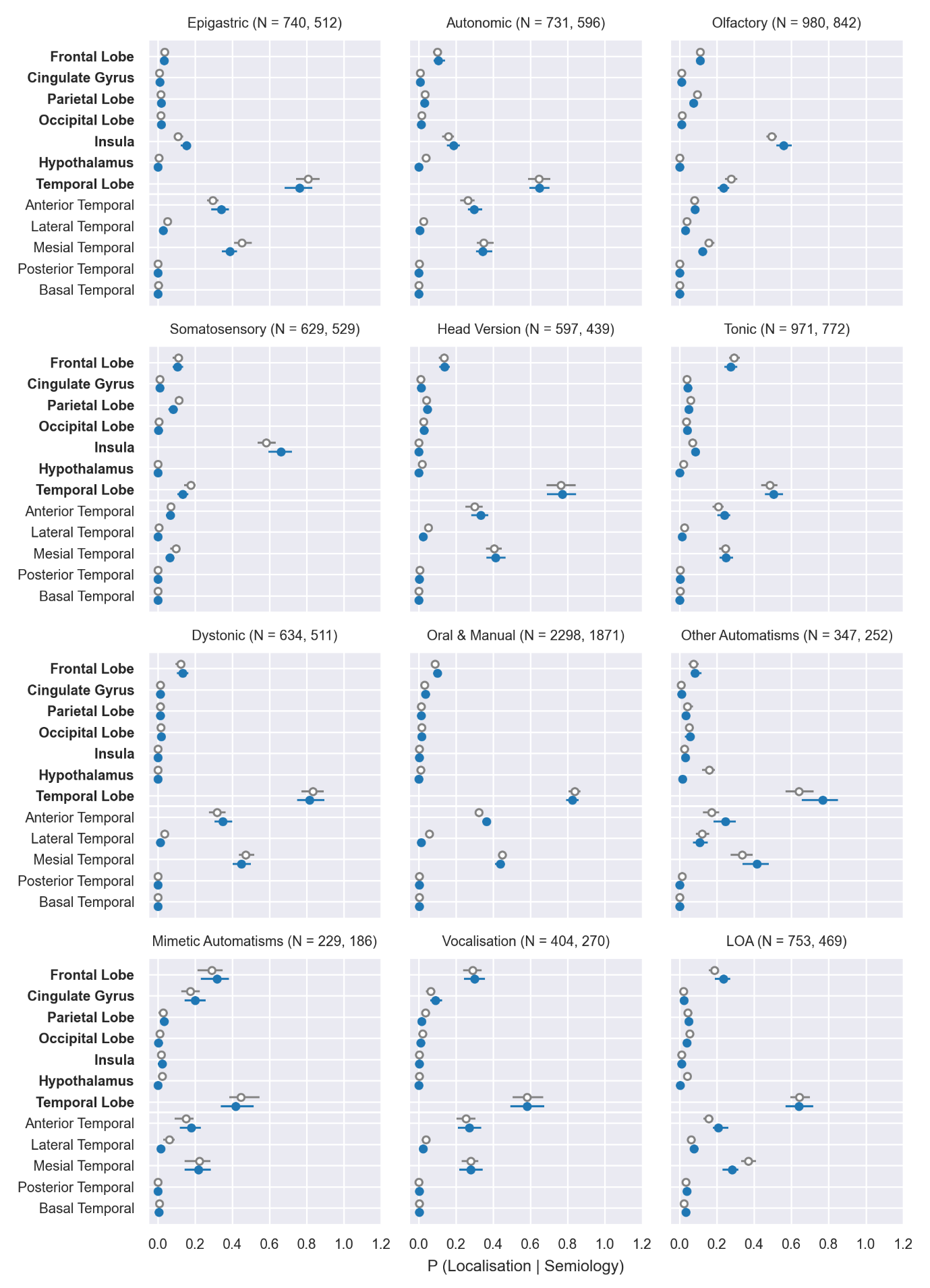


**Supplementary Figure 5 Ground-Truth Sensitivity Analysis with all-data (unfiltered):** **localising values for the 12 most commonly occurring semiologies.** Empty grey circles are all-three ground truths (same as empty grey circles in main manuscript fig. 3). Filled blue circles here are seizure-freedom ground-truth only for all-data (same as grey empty circles in supplementary fig. 4 above).


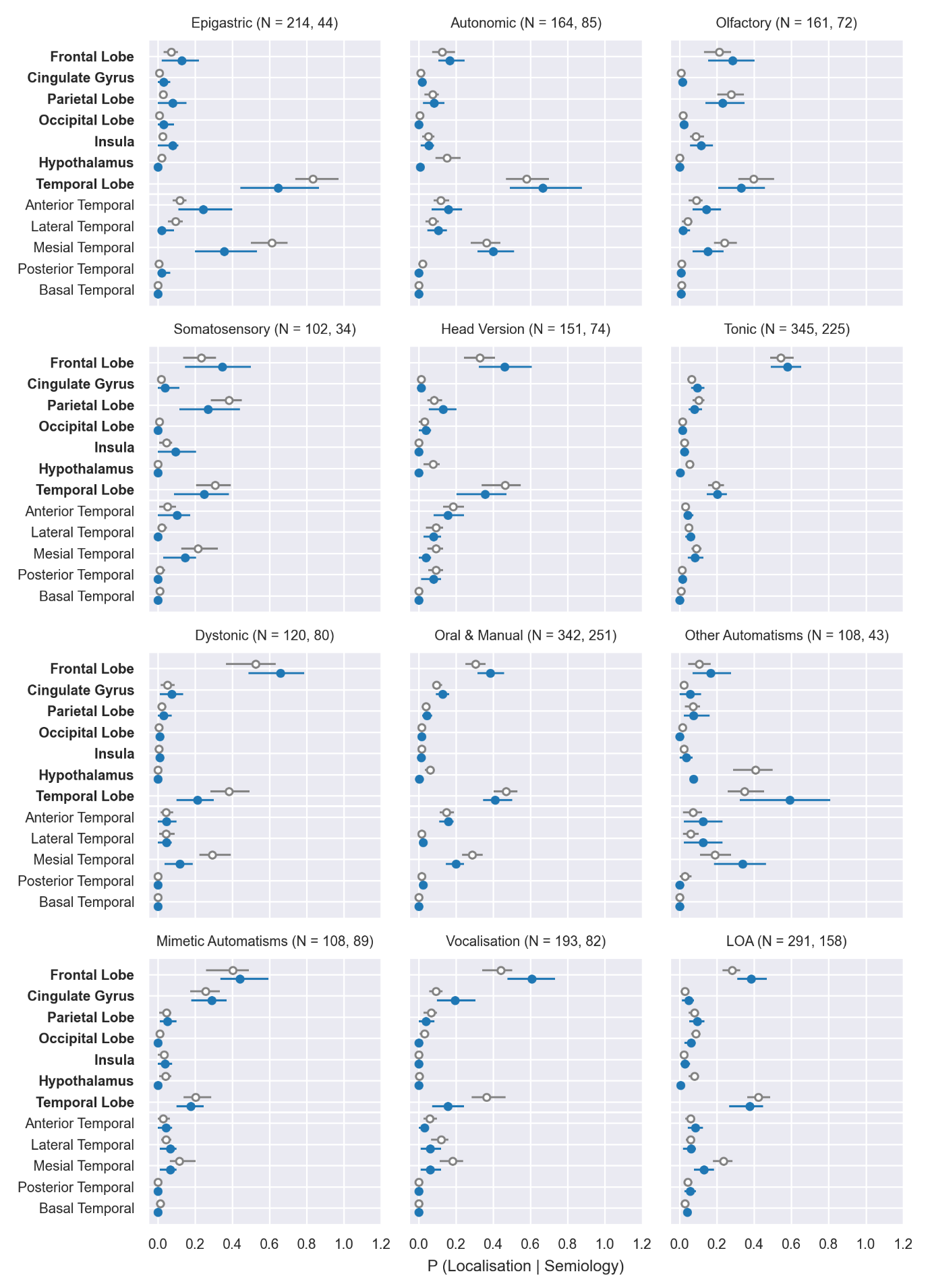


**Supplementary Figure 6 Ground-Truth Sensitivity Analysis with filtered-data:** **localising values for the 12 most commonly occurring semiologies.** Empty grey circles are all-three ground-truths from filtered-data (same as filled blue circles in main manuscript Fig. 3). Filled blue circles here are seizure-freedom ground-truth only from filtered-data (same as filled blue circles in supplementary Fig. 4 above).

### Age Labels: Adults Only


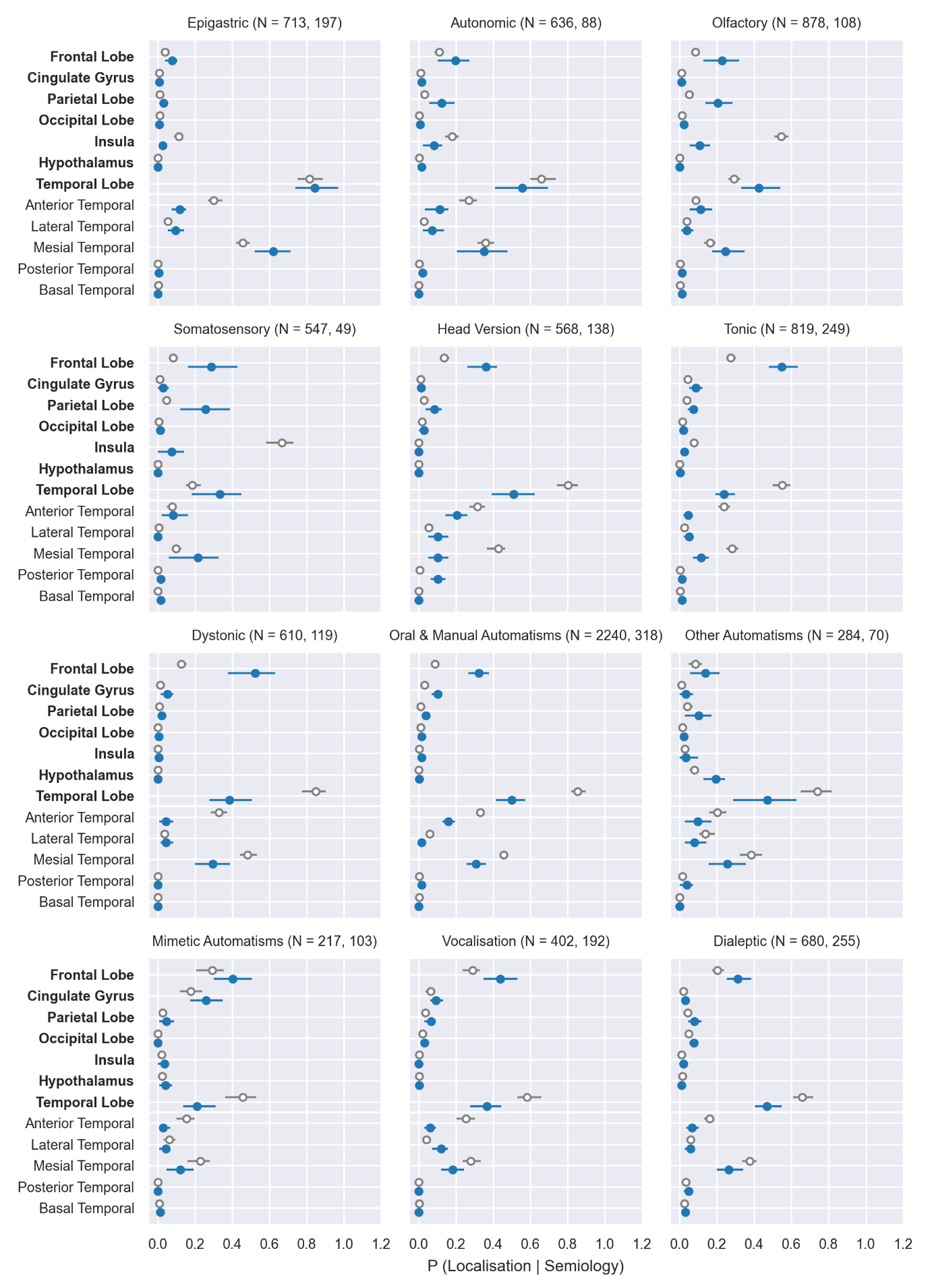


**Supplementary Figure 7: Seizure semiology localising values for the 12 most commonly occurring semiologies, excluding children under 7 years (where age labels are known).** This Figure is the counterpart to main manuscript figure 3, excluding datapoints with age labels under 7 years old (all ground-truths). Empty circles: all-data. Filled blue circles: topological filtered data.

# Supplementary Limitations

## Ground Truths

Studies such as SPECT are limited by the required imaging technology, rapid administration required, and poor inter-observer agreements for interpretation of SPECT images (kappa < 0.4)^3^ but were used for multimodal concordance in the database.

## Topological priors and Bayesian filtering

Our correction methods for publication bias involved selectively filtering the topological subset of the database, because non-topological studies more accurately portrayed the conditional probability of localisation to a lobe given a semiology. However, as seen in the case of the insula which disproportionately features datapoints from topological studies, some regions may require a high degree of prior clinical suspicion in order to be detected.

This highlights a limitation in simply filtering out topological studies. As topological studies could be modelled to represent the probability of a semiology occurring given a brain region, instead of excluding data, in future a Bayesian formulation of the conditional probabilities from topological studies could be helpful in leveraging these datapoints to invert the conditional probabilities and predict seizure foci. A subsequent clinical validation on an inverse variance weighted average of the non-topological values used in our main manuscript with a Bayesian formulation of the topological studies could be used.

As the *Semio2Brain* database is at best a sample of all semiologies taken from the literature, and even though we mitigated topological localising bias, it is possible to still have unmitigated semiological biases. This precluded entirely excluding the relative values of brain regions with odds ratios below 1 as the source of seizures.

## Descriptions of Semiology and Localisations

The collected semiologies are neither all the initial semiology nor consistently the full set, rather a hybrid due to reporting bias of prominent semiologies even though they may occur late in the sequence. Nevertheless, this is likely to make the database and results more clinically applicable, as many auras are experiential and may not have objective correlates and even if recollected, only the most prominent amongst many may be reported.^4^

Seizure propagation may be unidirectional, emphasising the value of sequences over sets, e.g., caudal spread from prefrontal cortex but not vice versa.^5^ Therefore, semiological sequences are likely to have better localising values to early propagating networks compared to our dataset because *Semio2Brain* is devoid of chronology.

## Normalising to Patient Numbers

In normalising to the number of patients as described in the main manuscript, we assumed that multiple localising datapoints are distributed equally between multiple patients per database row, because we used a simple normalising ratio.

# Supplementary References

1. Cardoso MJ, Modat M, Wolz R, et al. Geodesic information flows: spatially-variant graphs and their application to segmentation and fusion. *IEEE transactions on medical imaging.* 2015;34(9):1976-1988.

2. Kikinis R, Pieper SD, Vosburgh KG. 3D Slicer: A Platform for Subject-Specific Image Analysis, Visualization, and Clinical Support. In: Jolesz FA, ed. *Intraoperative Imaging and Image-Guided Therapy.* New York, NY: Springer New York; 2014:277-289.

3. Elwan SA, Wu G, Huang SS, Najm IM, So NK. Ictal single photon emission computed tomography in epileptic auras. *Epilepsia.* 2014;55(1):133-136.

4. Hirfanoglu T, Serdaroglu A, Cansu A, Bilir E, Gucuyener K. Semiological seizure classification: before and after video-EEG monitoring of seizures. *Pediatr Neurol.* 2007;36(4):231-235.

5. Bonini F, McGonigal A, Trebuchon A, et al. Frontal lobe seizures: from clinical semiology to localization. *Epilepsia.* 2014;55(2):264-277.
